# An algorithm to identify less invasive surfactant administration using a real-world database of preterm infants

**DOI:** 10.1371/journal.pone.0345768

**Published:** 2026-04-15

**Authors:** Xuezheng Sun, Annie N. Simpson, Aditi Lahiri, Sanjida Mowla, Dana Edelman, Shelby Corman, Daniel Fuentes, Dalibor Kurepa, Michael W. Kuzniewicz

**Affiliations:** 1 Chiesi USA Inc, Cary, North Carolina, United States of America; 2 Precision AQ, Bethesda, Maryland, United States of America; 3 Division of Research, Kaiser Permanente Northern California, Pleasanton, California, United States of America; 4 Cincinnati Children’s Hospital Medical Center, University of Cincinnati College of Medicine, Cincinnati, Ohio, United States of America; 5 Department of Pediatrics, University of California, San Francisco, California, United States of America; 6 The Permanente Medical Group, Oakland, California, United States of America; Ibaraki Children#39;s Hospital: Ibaraki Kenritsu Kodomo Byoin, JAPAN

## Abstract

**Background:**

Surfactant replacement therapy is central to respiratory distress syndrome (RDS) management in preterm infants, which can be delivered using a variety of methods. Less invasive surfactant administration (LISA) has been increasingly adopted due to its association with improved neonatal outcomes. However, there are no procedure codes to identify LISA in large real-world data (RWD), limiting the ability to evaluate its use and effectiveness on a large scale. This study aimed to develop an algorithm to identify LISA procedures using administrative data.

**Methods:**

We conducted a retrospective study using chart reviews as the gold standard to identify preterm infants receiving surfactant via LISA or non-LISA procedures across Kaiser Permanente Northern California (KPNC) facilities. We selected 82 candidate variables from administrative data between birth and date of first surfactant administration. The algorithm was developed using births between 2019 and 2023, which were randomly split into a training set (n = 884) and testing set (n = 379). A least absolute shrinkage and selection operator (LASSO) regression was used for variable selection and model fitting. Model discrimination was evaluated using area under the receiver operating characteristic (AUROC). Algorithm performance was validated using a combined sample of the testing set and a 2024 birth cohort (n = 622) overall and by gestational age (GA) using sensitivity (Sn), specificity (Sp), positive predictive value (PPV) and negative predictive value (NPV).

**Results:**

Among 1,263 preterm infants who received surfactant, 462 (36.6%) received surfactant via LISA and 801 (63.4%) received surfactant via invasive modalities (ETT or INSURE). The LASSO-based model selected 21 variables predictive of LISA methods based on the training set. The model demonstrated strong discrimination (AUROC = 0.87). Using the maximum specificity cut-point (predicted probability ≥0.79), the model achieved Sn = 43.9%, Sp = 96.8%, PPV = 90.0% and NPV = 72.5%, with an overall agreement of 75.9% when evaluated in the combined testing set and 2024 birth cohort. Sn and Sp were consistent across GA subgroups.

**Conclusions:**

We used a machine-learning approach to develop an algorithm that performed well in identifying surfactant administered via LISA among preterm infants using administrative data. The algorithm demonstrated strong performance and can support future research to evaluate the utilization and outcomes of LISA using RWD.

## Introduction

Respiratory distress syndrome (RDS) is a common and serious complication of preterm birth characterized by limited surfactant production due to lung immaturity [1]. RDS affects approximately 24,000 births annually in the US, with a substantial proportion of preterm births impacted [2]. Surfactant replacement therapy plays a key role in the management of RDS, improving survival by limiting the risk of clinical complications and the need for invasive ventilation [3]. Although surfactant therapy is most commonly used to manage RDS, it is also used in clinical practice for other neonatal respiratory conditions and off-label use has been described in multiple settings [4]. Until recently, surfactant was typically administered via endotracheal intubation and mechanical ventilation, more invasive approaches associated with increased risks of lung injury and clinical complications. To decrease these risks and the duration of mechanical ventilation, there has been a shift toward less invasive methods.

Surfactant administered via a thin catheter to infants on CPAP is often referred to as Less Invasive Surfactant Administration (LISA) or Minimally Invasive Surfactant Therapy (MIST) [5]. Although terminology varies by region, these approaches describe similar techniques. LISA methods are becoming preferred and are currently the recommended approach by the European Consensus Guidelines on the Management of Respiratory Distress Syndrome if an infant remains stable on CPAP while RDS symptoms worsen [6,7]. Additionally, since these less invasive methods avoid the need for intubation and mechanical ventilation, they are associated with fewer adverse clinical outcomes [8–11]. While use of LISA across the US has been more gradual compared to other regions, recent surveys indicate that utilization has more than doubled since 2018, although there remain barriers that limit greater implementation [12,13].

As LISA methods become more widely adopted, it is important to understand its real-world applications and impact on clinical outcomes. However, identifying LISA in routinely collected data is challenging because there is currently no specific billing, diagnostic or procedure codes for this method in administrative claims data or structured EHR data. These limitations prevent our ability to evaluate how widely LISA is being implemented, monitor variation in practice across facilities, and assess the effectiveness and safety across surfactant administration modalities in large, diverse populations. Determining this evidence at the population level is essential to inform efforts to improve neonatal care, guide policy decisions and advance respiratory management for preterm infants.

To improve our understanding of clinical management of preterm infants, the goal of this study was to develop a code-based algorithm to identify administration of surfactant via LISA methods using routinely collected administrative data and a machine learning approach. This study was designed as a methodological evaluation to reliably identify LISA in real-world data and was not intended to evaluate the clinical effectiveness or safety of LISA, which has been addressed in previous studies [11,14]. By developing an algorithm, this work provides a foundation for future real-world studies to characterize utilization patterns and to compare outcomes across surfactant administration modalities as the use of less invasive methods increase.

## Methods

### Data source and study population

This study analyzed a retrospective cohort to identify modalities by which surfactant was administered to preterm infants. This study utilized comprehensive, real-world data from Kaiser Permanente Northern California (KPNC) healthcare system serving nearly 4.5 million members across 15 birth facilities. KPNC’s integrated electronic medical record (EMR) database includes information on enrollment, demographics, census-level data, procedures and diagnoses, pharmacy data, laboratory reports, hospitalizations, emergency department visits and mortality. Administrative data were extracted from one of the components included in KPNC’s EMR, which includes standardized billing codes (ICD-10, CPT, HCPCS, NDC) collected from birth through the date of first surfactant administration.

The study population included liveborn preterm infants (<37 weeks) delivered at KPNC facilities between January 1, 2019, and December 31, 2023, who received surfactant during birth hospitalization. Exclusion criteria included infants with congenital anomalies, those receiving respiratory support for non-RDS indications, and those who died in the delivery room.

### Statistical analyses

#### Population characteristics.

We present characteristics for the overall study population across training and testing cohorts and by modality (LISA vs. non-LISA) using univariate analyses. Categorical variables were summarized with counts and percentages, while continuous variables were summarized using measures of central tendency (e.g., mean and standard deviation or median and interquartile range, as appropriate).

#### Reference standard for LISA identification.

The modality of surfactant administration was determined using detailed chart review conducted by trained clinical specialists, which served as the gold standard. Modality was identified from procedures which documented the date, time, and modality of surfactant administration. We focused on modality of first successful surfactant administration, though some infants may have received multiple administrations. In cases where procedure notes were unavailable, additional clinical documentation was reviewed to determine modality. After the initial reviewer made assessments, a second reviewer independently evaluated 20% of charts and any discrepancies were reconciled by a third reviewer. Surfactant administration was classified as a dichotomous variable as either LISA administered using a thin catheter or non-LISA methods which include invasive methods (endotracheal intubation [ETT] or intubate-surfactant-extubate [INSURE]). Clinical indications for surfactant administration and specific treatment thresholds are not available in administrative data and may vary by clinician and facility. Overall, this study focuses on identifying the mode of surfactant administration (LISA vs. non-LISA) rather than the clinical indication of treatment.

#### Algorithm development (LASSO derived prediction model in training set).

We developed and validated an algorithm to identify LISA procedures in real-world data using administrative data. We selected 82 candidate variables based on expert opinion and literature review, (**S1 Table**), which include demographic, maternal and infant characteristics and details of respiratory support and medical treatment before and after surfactant administration.

Of the sample of preterm infants born during 2019–2023, 70% were randomly selected and composed a training set (n = 884). First, the algorithm development included candidate variable selection as described above. Second, we used least absolute shrinkage and selection operator (LASSO) regression, a machine-learning approach, for variable selection and coefficient shrinkage to predict LISA procedures. The optimal model was selected based on the lowest average Mean Square Error (MSE) using k-fold cross-validation. The model discrimination was evaluated using the area under the receiver operating characteristics (AUROC) to determine a cut-point indicating surfactant receipt via LISA methods. We calculated accuracy metrics including sensitivity, specificity, positive predictive value (PPV) and negative predictive value (NPV) by comparing algorithm predictions for LISA modality against the chart review gold standard.

#### Algorithm validation in the combined testing set and 2024 cohort.

We assessed model performance in a combined testing set (n = 622), which included the remaining 30% of the 2019−2023 cohort (n = 379) and a sample of births during 2024 (n = 243). Model performance was also evaluated within the combined testing set and subgroups by gestational age (<34 weeks and ≥34 weeks). We evaluated overall discrimination using the AUROC, which summarizes the model’s ability to differentiate between infants who did and did not receive surfactant via LISA. Continuous prediction scores were used to calculate probabilities of administration via LISA to classify infants as receiving LISA or not based on specific cut-points. We identified optimal cut points for continuous prediction scores using a cut point that maximized specificity to explore performance trade-offs when prioritizing reduced false positives and using Youden’s Index, optimizing sensitivity and specificity (sensitivity + specificity −1) [15,16].

#### Ethics approval.

The study protocol was reviewed by the KPNC Institutional Review Board (IRB), which granted an exemption and approved a waiver of informed consent and authorization as the study involved analysis of existing data and records without direct patient identifiers (IRB # 00001045). Therefore, informed consent from individual participants was not required.

## Results

### Patient characteristics

Among the 1,263 preterm infants who received surfactant at KPNC facilities between 2019 and 2023, 884 were included in the training cohort (**Table 1**). Overall, the training cohort included a higher proportion of males (61.3%), infants delivered by cesarean section (74.9%) and born at level 3 facilities (85.2%). Additionally, almost 20% of infants were from multiple pregnancies. The majority of mothers received antenatal steroids (66.6%) and most infants survived to discharge (88.8%).

**Table 1:** Demographic characteristics for training cohort.

	Training Cohort (N = 884)			
Characteristic	Total, N = 884	Non-LISA, N = 563	LISA, N = 321	p-value^1^
**Sex, n (%)**				>0.9
*Female*	342 (38.7%)	217 (38.5%)	125 (38.9%)	
*Male*	542 (61.3%)	346 (61.5%)	196 (61.1%)	
**Multiple pregnancy, n (%)**	172 (19.5%)	110 (19.5%)	62 (19.3%)	>0.9
**Birth facility level, n (%)**				0.6
*1*	13 (1.5%)	8 (1.4%)	5 (1.6%)	
*2*	118 (13.4%)	80 (14.2%)	38 (11.8%)	
*3*	753 (85.2%)	475 (84.4%)	278 (86.6%)	
**Type of delivery, n (%)**				0.2
*Cesarean*	662 (74.9%)	429 (76.2%)	233 (72.6%)	
*Vaginal*	222 (25.1%)	134 (23.8%)	88 (27.4%)	
**SGA, n (%)**	74 (8.4%)	45 (8.0%)	29 (9.0%)	0.6
**LGA, n (%)**	109 (12.3%)	76 (13.5%)	33 (10.3%)	0.2
**Gestational age (weeks), n (%)**				<0.001
*23-27*	309 (35.0%)	249 (44.2%)	60 (18.7%)	
*28-31*	295 (33.4%)	158 (28.1%)	137 (42.7%)	
*32-36*	280 (31.7%)	156 (27.7%)	124 (38.6%)	
**Birth year, n (%)**				<0.001
*2019*	150 (17.0%)	123 (21.8%)	27 (8.4%)	
*2020*	155 (17.5%)	102 (18.1%)	53 (16.5%)	
*2021*	181 (20.5%)	111 (19.7%)	70 (21.8%)	
*2022*	175 (19.8%)	104 (18.5%)	71 (22.1%)	
*2023*	223 (25.2%)	123 (21.8%)	100 (31.2%)	
**Final disposition, n (%)**				<0.001
*Alive/Home*	785 (88.8%)	475 (84.4%)	310 (96.6%)	
*Death*	99 (11.2%)	88 (15.6%)	11 (3.4%)	
**ICD TTN, n (%)**	88 (10.0%)	48 (8.5%)	40 (12.5%)	0.060
**ICD RDS, n (%)**	807 (91.3%)	513 (91.1%)	294 (91.6%)	0.8
**ICD RDS other, n (%)**	26 (2.9%)	19 (3.4%)	7 (2.2%)	0.3
**ICD RDS unspecified, n (%)**	482 (54.5%)	312 (55.4%)	170 (53.0%)	0.5
**Apgar 5 score < 7, n (%)**	241 (27.3%)	204 (36.2%)	37 (11.5%)	<0.001
**Highest FiO2 pre-surfactant, n (%)**				
Mean (SD)	–	62 (28)	50 (20)	<0.001
Median (IQR)	–	51 (40, 100)	44 (35, 60)	<0.001
Missing	65	63	2	
**Respiratory support pre-surfactant, n (%)**				<0.001
CVENT	172 (19.5%)	172 (30.6%)	0 (0.0%)	<0.001
HFNC	2 (0.2%)	0 (0.0%)	2 (0.6%)	
HFV	10 (1.1%)	10 (1.8%)	0 (0.0%)	
NCPAP	563 (63.7%)	263 (46.7%)	300 (93.5%)	
NIPPV	22 (2.5%)	7 (1.2%)	15 (4.7%)	
Unknown	115 (13.0%)	111 (19.7%)	4 (1.2%)	
**Maternal age**				
Mean (SD)	–	32.0 (6.0)	33.0 (5.6)	0.011
Median (IQR)	–	32.2 (28.0, 36.1)	33.4 (29.3, 36.7)	0.013
**Maternal race/ethnicity, n (%)**				0.4
*Asian*	195 (22.1%)	128 (22.7%)	67 (20.9%)	
*Black*	116 (13.1%)	81 (14.4%)	35 (10.9%)	
*Hispanic*	273 (31.0%)	166 (29.5%)	107 (33.3%)	
*Other/Missing*	40 (4.5%)	27 (4.8%)	13 (4.0%)	
*White*	260 (29.4%)	161 (28.6%)	99 (30.8%)	
**Antenatal steroid, n (%)**	589 (66.6%)	377 (67.0%)	212 (66.0%)	0.8

^Pearson’s Chi-squared test; Fisher’s exact test; Wilcoxon rank sum test; Fisher’s Exact Test for Count Data with simulated p-value (based on 2000 replicates).^

Within the training cohort, infants who received LISA were generally more clinically stable than those treated with non-LISA methods, as reflected by higher gestational age (81.3% vs. 55.8% born between 28–36 weeks), greater use of non-invasive respiratory support prior to surfactant administration (NCPAP: 93.5% vs. 46.7%; NIPPV: 4.7% vs 1.2%), higher Apgar scores and lower FiO_2_ before surfactant. Infants treated with LISA also had a higher rate of survival to discharge (96.6% vs. 84.4%) compared to infants who did not receive surfactant via LISA. Maternal characteristics including age, race/ethnicity and antenatal steroid exposure were comparable across groups. Diagnoses codes including TTN and RDS represent conditions documented during hospitalization and are not mutually exclusive. The inclusion of these conditions represents clinical context rather than the indication for surfactant administration. Similar patterns were observed among infants in the testing set (**S2 Table**).

### Algorithm development

Of the 82 candidate variables derived from administrative billings codes (**S1 Table**), 21 variables were retained in the final model using LASSO regression within the training dataset (n = 884; **S1 Fig** **and Table 2**). The model’s optimal performance corresponded to a coefficient of variation for the predicted residual error sum of squared (CV PRESS) of 119.77 (**S1 Fig**).

**Table 2:** Predictors of LISA.

Variable	Estimate	Pr > ChiSq
Extremely low birth weight newborn, 500–749 grams (Y/N)	−0.5946	0.0413
Preeclampsia (Y/N)	0.5541	0.0016
Newborn affected by Premature rupture of membrane (Y/N)	1.2344	0.0456
Extreme immaturity of newborn, gestational age 23 completed weeks (Y/N)	−2.2044	0.0074
Estimated fetal weight is greater than 90^th^ percentile (Large for Gestational Age)	−0.7344	0.0043
Extreme immaturity of newborn, gestational age 25 completed weeks (Y/N)	−0.4642	0.1575
Preterm newborn, gestational age 29 completed weeks (Y/N)	0.4784	0.0546
Preterm newborn, gestational age 32 completed weeks (Y/N)	0.207	0.4757
Preterm newborn, gestational age 36 completed weeks (Y/N)	0.4654	0.0829
Assistance with Respiratory Ventilation, Less than 24 Consecutive Hours, Continuous Positive Airway Pressure (Y/N)	0.5385	0.0831
Assistance with Respiratory Ventilation, Greater than 96 Consecutive Hours, Continuous Positive Airway Pressure (Y/N)	−0.7522	0.0005
Assistance with Respiratory Ventilation, 24–96 Consecutive Hours, Continuous Positive Airway Pressure (Y/N)	−0.9815	0.0116
Assistance with Respiratory Ventilation, Less than 24 Consecutive Hours, High Nasal Flow/Velocity (Y/N)	1.0398	0.0024
Insertion of Endotracheal Airway into Trachea, Via Natural or Artificial Opening (Y/N)	−0.8193	0.0004
Respiratory Ventilation, Less than 24 Consecutive Hours (Y/N)	−0.7745	0.0973
Introduction of Other Therapeutic Substance into Respiratory Tract, Via Natural or Artificial Opening (Y/N)	0.9728	<.0001
Fentanyl administered on same calendar day of surfactant administration	−2.6868	<.0001
Morphine administered on same calendar day of surfactant administration	−0.7964	0.0063
Atropine administered on same calendar day of surfactant administration	3.4235	<.0001
Neuromuscular relaxant administered on same calendar day of surfactant administration	−0.4038	0.5351
Asian race	−0.2856	0.2031

The selected predictors of LISA included indicators of gestational age and birth weight, respiratory support, clinical factors including medication use and procedure timing (**Table 2**). Together, these variables characterize the typical clinical profile of infants treated with LISA procedures. Compared to those who received surfactant via non-LISA modalities, who often require intubation or more advanced respiratory support at the time of administration, infants who were treated with LISA received non-invasive respiratory support, most commonly including CPAP for <24 hours and HFNC, prior to surfactant administration.

**Fig 1:**
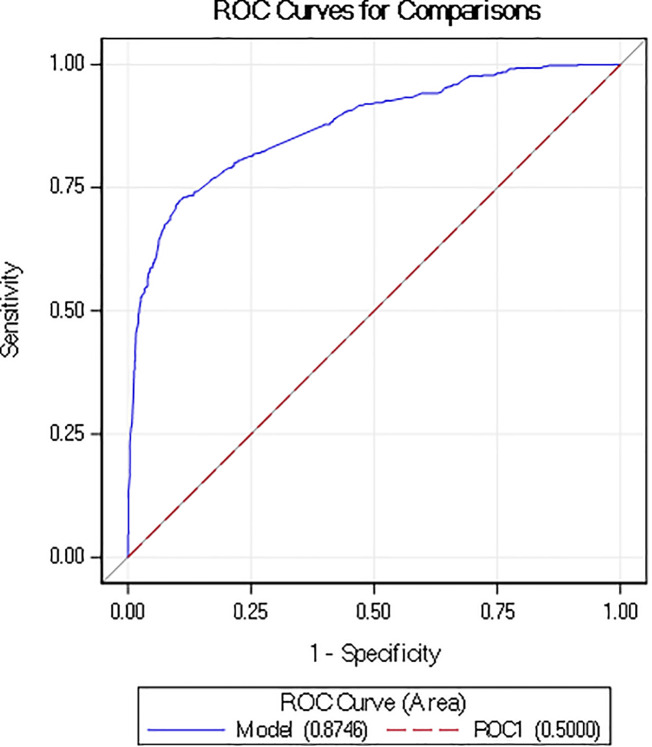
ROC AUC.

Among the retained predictor variables, atropine use was the strongest positive predictor of LISA procedures (OR=30.7; p < 0.0001; **Table 2**). In contrast, infants requiring prolonged respiratory support, ETT or medication use including fentanyl and morphine on the day of surfactant administration had lower odds of receiving LISA. Additionally, infants born at 23 weeks’ gestation and those with extremely low birth weight (500−749 grams) were less likely to receive surfactant via LISA. Maternal and demographic characteristics contributed minimally to model performance. Overall, the model demonstrated strong discrimination for identifying LISA, with an AUROC of 0.87 (95% CI: 0.85–0.89; **Fig 1**).

### Algorithm performance in training set

The model performance was evaluated in the overall training cohort (n = 884) and by gestational age using both the maximum specificity cut point and Youden’s cut point to determine the optimal threshold for classifying LISA procedures.

**Fig 2:**
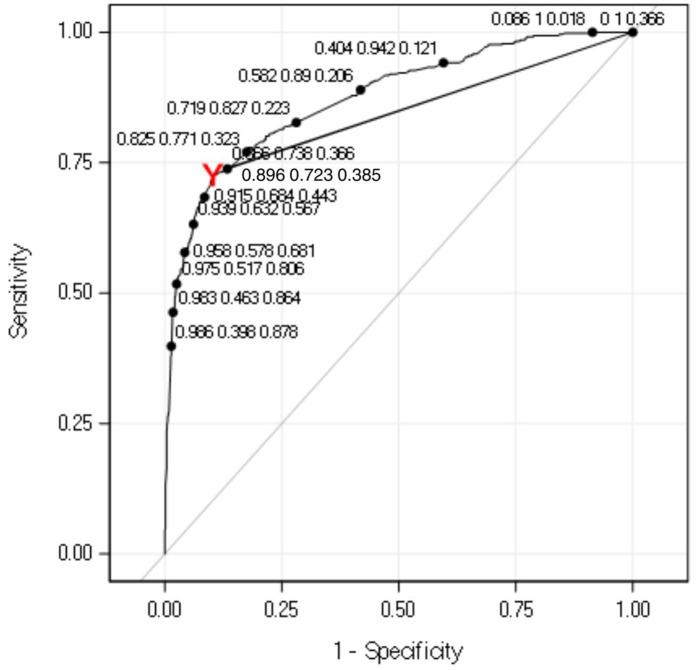
ROC AUC with selected cut points in bold (labeled with specificity, sensitivity, and cut point value).

In the overall training cohort, using the maximum specificity cut point (predicted probability ≥ 0.79), the model achieved a Sn = 41.1% (95% CI: 35.7–46.7) and Sp = 99.1 (95% CI: 97.9–99.7), with an accuracy of 78.1% (**S3 Table**). When using Youden’s cut point (**Fig 2**; predicted probability ≥ 0.38), the model achieved Sn = 71.3% (95% CI: 66.1–76.2) and Sp = 90.6 (95% CI: 87.8–92.9), with an overall accuracy of 83.6% (**S4 Table**). While the model which maximizes specificity resulted in a reduced sensitivity, this model showed a greater ability to correctly identify true negatives, minimizing false positives in this population.

Among infants born at <34 weeks’ gestation, performance was comparable to that observed in the overall cohort. Under the maximum specificity cut point, the model resulted in Sn = 40.5% (95% CI: 34.3–46.9) and Sp = 99.4% (95% CI: 98.2–99.9) with an accuracy of 79.3% (**S3 Table**). Using Youden’s cut point, the model shows a Sn = 73.3% (95% CI: 67.3–78.7) and Sp = 91.0% (95% CI: 88.1–93.4) with an accuracy of 84.9% (**S4 Table**). Among infants born ≥34 weeks’ gestation, performance was lower when compared to the overall cohort. When using the maximum specificity cut point, the model achieved Sn = 43.2% (95% CI: 31.8–55.3) and Sp = 97.7% (95% CI: 91.8–99.7), with an accuracy of 72.3% (**S3 Table**). Using Youden’s cut point resulted in Sn = 64.9% (95% CI: 52.9–75.6) and Sp = 88.2% (95% CI: 79.4–94.2), with an accuracy of 77.4% (**S4 Table**).

The maximum specificity cut point was selected to determine the primary model when validating the algorithm because of its clinical interpretability and alignment with our goal to minimize false positive predictions of infants who receive surfactant via LISA procedures. By prioritizing high specificity, we aimed to ensure that infants classified as receiving LISA are highly likely to have truly received surfactant via this method, allowing us to define a high-confidence LISA cohort. This approach is important for future comparative effectiveness or safety studies where outcome estimates may be biased if other surfactant administration methods are misclassified as LISA.

### Algorithm validation in testing plus 2024 cohort

The algorithm derived using variables identified through LASSO regression was evaluated in the combined testing and 2024 validation dataset (n = 622) and demonstrated strong overall performance. Using the maximum specificity cut point, sensitivity was 43.9% (95% CI: 37.6–50.4) and specificity was 96.8% (95% CI: 94.5–98.3), consistent with findings in the training cohort and full dataset. The algorithm maintained strong discrimination between LISA and non-LISA procedures with high specificity (**Table 3**).

**Table 3:** Algorithm validation in testing + 2024 birth cohort overall and by gestational age using maximized specificity cut point.

Statistic	Overall	GA < 34 weeks	GA ≥ 34 weeks
Number of infants (N)	622	494	128
Sensitivity, % (95% CI)	43.9 (37.6–50.4)	39.8 (32.8–47.1)	58.2 (44.1–71.4)
Specificity, % (95% CI)	96.8 (94.5–98.3)	97.0 (94.4–98.6)	95.9 (88.5–99.1)
Positive predictive value, % (95% CI)	90.0 (83.5–94.1)	89.4 (81.3–94.3)	91.4 (77.5–97.1)
Negative predictive value, % (95% CI)	72.5 (70.2–74.7)	71.9 (69.5–74.2)	75.3 (68.6–80.7)
Accuracy, % (95% CI)	75.9 (72.3–79.2)	74.9 (70.8–78.7)	79.7 (71.7–86.3)
Positive likelihood ratio	13.8	13.4	14.2
Negative likelihood ratio	0.58	0.62	0.44
Estimated disease prevalence, % (95% CI)	39.6 (35.7–43.5)	38.7 (34.4–43.1)	42.9 (34.3–52.0)

When stratified by gestational age, the algorithm’s performance was consistent (**Table 3**). Among infants born at <34 weeks gestation (n = 494), the model achieved a sensitivity of 39.8% (95% CI: 32.8–47.1) and specificity of 97.0% (95% CI: 94.4–98.6), while among those born at ≥34 weeks (n = 128), sensitivity was 58.2% (95% CI: 44.1–71.4) and specificity was 95.9% (95% CI: 88.5–99.1). These results suggest that the algorithm performed consistently across gestational age subgroups, with slightly higher sensitivity observed among more mature infants.

When using an alternative cut point based on Youden’s Index (p ≥ 0.39) for the combined testing and 2024 validation cohort, the algorithm demonstrated a sensitivity of 73.9% and specificity of 79.8% (**S5 Table**). When stratified by gestational age, the model performed similarly among infants <34 weeks (Sn = 70.7%, Sp = 82.5%) but performance was lower among those ≥34 weeks (Sn = 85.5%, Sp = 68.5%), likely a reflection of the smaller sample (**S5 Table**). Compared to the higher threshold that maximized specificity in our first approach, this approach provided more balance across sensitivity and specificity. Overall, this method identified a greater proportion of true LISA cases though potentially increasing false positives, which highlights the tradeoff across the different approaches.

## Discussion

This study developed and validated an algorithm using machine-learning methods to identify preterm infants who received surfactant via LISA using routinely collected administrative data from a large, integrated US healthcare system. By utilizing combinations of diagnostic and procedural codes, the algorithm demonstrated high discrimination and specificity across training and validation cohorts, including gestational age subgroups. These observations indicate that machine-learning approaches can reliably identify LISA procedures in real-world administrative data, offering a novel approach for monitoring LISA adoption and implementation across large and diverse clinical populations.

While LISA has been widely used across many European countries, its adoption in the U.S. has been more limited, though gradually increasing over the years. A 2018 national survey of US neonatologists found that only 15% of 472 respondents reported using LISA in either routine care or in research settings [12]. A more recent 2025 survey evaluated the rate of utilization of MIST [13]. Of the 381 respondents, most of which were neonatologists, 39% used MIST. This reflects a substantial increase in adoption over time, although utilization varied across different healthcare facilities and regions [13]. These surveys indicate a growing interest in thin catheter procedures across US NICUs while highlighting barriers to adoption, including limited provider training and wide variation in clinical protocols [12,13].

Despite the growing use of LISA, substantial research gaps remain in understanding real-world patterns of LISA utilization and its impact across diverse neonatal populations. Although randomized trials and meta-analyses demonstrate that LISA reduces the need for mechanical ventilation, lowers the risk of bronchopulmonary dysplasia (BPD), and improves short term outcomes compared with traditional intubation-based approaches, these studies are typically small, include heterogeneous patient populations and are conducted in limited settings [10,11,17]. Observational cohorts from Europe, including a large cohort study of the German Neonatal Network (GNN) and a smaller study based in Spain, show associations between LISA and lower rates of mortality, BPD, intraventricular hemorrhage and retinopathy of prematurity [8,18]. A recent single-center retrospective cohort study evaluated the use of a standardized respiratory care protocol incorporating LISA in preterm infants and evaluated factors associated with its success or failure [19]. The study observed that LISA reduced the need for mechanical ventilation and identified gestational age and early oxygen requirements as key predictors of failure [19]. Despite these insights, little is known about how US infants are selected to receive surfactant via LISA procedures, how consistently the procedure is performed and how outcomes vary across diverse clinical populations.

As RDS remains one of the most common and resource intensive complications among preterm infants, there is an increasing need for large-scale datasets to evaluate the real-world patterns of management of RDS. European guidelines now recommend LISA as the preferred modality for surfactant administration for spontaneously breathing infants, reflecting adherence towards approaches that minimize mechanical ventilation to improve short- and long-term respiratory outcomes [6]. As US practice begins to shift, population-level databases can provide important insights that small trials or meta-analyses cannot, including regional variations in practice and understanding how to plan for the increasing prevalence of RDS and expenses associated with ventilation use and NICU care. Additionally, large cohorts are essential in having the power to detect rare adverse events and to evaluate safety in subgroups including differences across preterm infants who may respond differently to less invasive methods. Larger databases allow for more robust epidemiologic analyses including adjusting for confounding and identifying trends over time that would be helpful to supplement previous findings from randomized trials or meta-analyses. However, because there are no standard procedure codes for LISA, these analyses would require validated tools, like machine-learning based algorithms, to accurately identify LISA within large administrative datasets.

### Strengths and limitations

There are strengths to the development and validation of this algorithm, including the availability of comprehensive electronic healthcare data that has consistently been collected and maintained within the KPNC healthcare system. This integrated system, which includes 15 birth facilities and serves almost a third of Northern California residents, allows for a large, diverse cohort with detailed clinical documentation, which enhances the accuracy of identifying LISA procedures among preterm infants and further supports the development of this algorithm.

However, there are several limitations to consider. First, as a retrospective analysis conducted within a single integrated health system with standardized protocols and documentation practices, the performance of this algorithm may not generalize to settings with different patient populations, documentation practices and administrative coding procedures. Some variables used in our model, including maternal characteristics, may not be readily available in other databases. Although misclassification is likely minimized within KPNC due to established data quality procedures, study variables may still be impacted by clinical coding errors and missing information.

Second, standardized clinical thresholds for surfactant initiation were not available in administrative data and may vary by clinician and facility. Therefore, we could not evaluate treatment indications. Although LISA has been widely used across Europe for several years, LISA and surfactant use for indications beyond RDS remain off label in the US [4]. Our objective was to identify the method of administration rather than distinguish on-label vs. off-label use. While off-label in the US, LISA has been used at KPNC since 2017 under established protocols, suggesting that this practice is not new but remains difficult to study at a larger scale because it typically requires manual chart review. In this context, our algorithm provides an efficient and reproducible approach to identifying LISA use in large administrative datasets and provides a foundation for future real-world studies of utilization and outcomes.

Third, atropine administration was a strong predictor in our model. However, premedication practices may vary across healthcare systems, which may impact algorithm performance in settings where atropine is not routinely used for LISA procedures. In addition, surfactant administration in the US is not limited to a single indication, and off-label use for conditions beyond RDS have been described in clinical practice [4]. In our study, diagnosis codes such as transient tachypnea of the newborn (TTN) were included as descriptive clinical characteristics and were not mutually exclusive with RDS diagnoses or used to determine the indication of surfactant treatment. Our model is data-driven and was designed to identify administration modality rather than clinical decision making.

Finally, this algorithm focuses on identifying LISA vs. non-LISA methods of first surfactant administration and does not distinguish between initial and subsequent doses. We also did not include other minimally invasive methods of administration (LMA-based approaches), and performance may differ in populations where these methods are more common. Future studies that incorporate more detailed clinical data could extend this approach to evaluate indications for treatment, repeat dosing and distinction among other minimally invasive techniques.

## Conclusion

As LISA adoption increases across the US and clinical practices continue to evolve, accurate identification of LISA within large databases is critical for understanding its real-world utilization for improving neonatal care. The machine-learning algorithm developed in this study provides a practical and reliable method for identifying LISA in administrative data, establishing a foundation for future work aimed at characterizing patterns of LISA utilization and comparing outcomes across surfactant administration modalities as the use of less invasive methods increase. This study developed and validated an algorithm using a large, integrated healthcare system with detailed clinical documentation, allowing for accurate identification of LISA in routinely collected data.

Future research should prioritize external validation across diverse healthcare systems to evaluate performance in settings with different documentation practices and LISA protocols, as well as ongoing evaluation as clinical approaches to less invasive methods continue to evolve. Although the algorithm performance may be lower in settings that rely primarily on claims data or have different coding and documentation practices, the algorithm is most likely to perform well in integrated health systems with similar study populations, structured procedure coding, and detailed administration records. Future work should evaluate how well the algorithm performs in other health systems and regions outside of Northern California. Until more standardized documentation of practices or procedure codes become widely available, this machine-learning based algorithm offers a valuable tool to support population-level studies evaluating effectiveness, safety and resource utilization across surfactant administration modalities and to inform evidence-based neonatal care.

This evidence is essential to inform clinicians, respiratory therapists and health systems as less invasive approaches continue to evolve, particularly in the absence of established procedure codes for LISA.

## Supporting information

S1 Fig Progression of ASE for LISA. (DOCX)

S1 Table Candidate variables. (DOCX)

S2 Table Demographic characteristics for testing cohort. (DOCX)

S3 Table Algorithm performance in training cohort overall and by gestational age using maximized specificity cut point. (DOCX)

S4 Table Algorithm performance in training cohort overall and by gestational age using Youden’s cut point. (DOCX)

S5 Table Algorithm validation in testing + 2024 birth cohort overall and by gestational age using Youden’s cut point. (DOCX)
