# Supplementary material for: An algorithm to identify less invasive surfactant administration using a real-world database of preterm infants
Source: PLoS One. 2026 Apr 15;21(4):e0345768. doi: 10.1371/journal.pone.0345768 (PMC13082626; doi:10.1371/journal.pone.0345768)
Supplement: S1 Fig — (DOCX) [file pone.0345768.s001.docx]

**Supplementary Fig****ure 1. Progression of ASE for LISA**

**
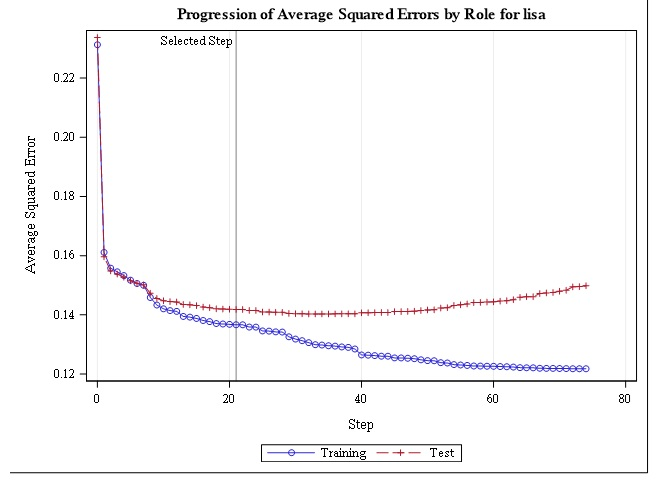
**

The optimal model was selected when the coefficient of variation for the predicted residual error sum of squares (CV PRESS) reached 119.77 at step 21.
