# Supplementary material for: An algorithm to identify less invasive surfactant administration using a real-world database of preterm infants
Source: PLoS One. 2026 Apr 15;21(4):e0345768. doi: 10.1371/journal.pone.0345768 (PMC13082626; doi:10.1371/journal.pone.0345768)
Supplement: S1 Table — (DOCX) [file pone.0345768.s002.docx]

**Supplemental Table 1. Candidate variables**

| **Variable Description** | **Categories or code Type** | **Code** |
| --- | --- | --- |
| Age | NA | NA |
| Infant Race | White  Black or African American  Asian  American Indian or Alaska Native  Native Hawaiian or Other Pacific Islander  Multiracial  Unknown/Other | NA |
| Ethnicity | Hispanic  Non-Hispanic  Missing/unknown | NA |
| Primary Payor | 1=Medicare or Medicaid  2=Commercial  3=Private  4=Self-funded  5=State subsidized  6=Other  7=Other KP  8=non-KPNC Medicare or Medicaid  9=non-KPNC other | NA |
|  |  |  |
| Substance use disorder (Y/N) | ICD-10-CM | F19 |
| Gestational diabetes (Y/N) | ICD-10-CM | O244 |
| Gestational hypertension (Y/N) | ICD-10-CM | O13 |
| Preeclampsia (Y/N) | ICD-10-CM | O14 |
| Eclampsia (Y/N) | ICD-10-CM | O15 |
| Chorioamionitis (Y/N) | ICD-10-CM | O4 |
| Premature rupture of membrane (Y/N) | ICD-10-CM | O42 |
| Newborn affected by maternal use of alcohol (Y/N) | ICD-10-CM | P043 |
| Newborn affected by maternal hypertensive disorders (Y/N) | ICD-10-CM | P000 |
| Newborn affected by other maternal conditions (Y/N) | ICD-10-CM | P0089 |
| Newborn affected by maternal use of unspecified drugs of addiction (Y/N) | ICD-10-CM | P0440 |
| Newborn affected by maternal use of cocaine (Y/N) | ICD-10-CM | P041 |
| Newborn affected by maternal use of tobacco (Y/N) | ICD-10-CM | P042 |
| Newborn affected by Chorioamionitis (Y/N) | ICD-10-CM | P027 |
| Estimated fetal weight is greater than 90^th^ percentile (Large for Gestational Age) | ICD-10-CM | O36.6x |
| Newborn affected by Premature rupture of membrane (Y/N) | ICD-10-CM | P011 |
| **Maternal characteristics** |  |  |
| Multiple pregnancy (Y/N) | ICD-10-CM | O30 |
| Vaginal delivery (Y/N) | CPT | 59400 |
| Caesarean delivery (Y/N) | CPT | 59510 |
| Encounter for Caesarean delivery without indication (Y/N) | ICD-10-CM | O82 |
| **Infant characteristics** |  |  |
| Respiratory distress syndrome of newborn (Y/N) | ICD-10-CM | P220 |
| Transient tachypnea of newborn (Y/N) | ICD-10-CM | P221 |
| Other respiratory distress of newborn (Y/N) | ICD-10-CM | P228 |
| Respiratory distress of newborn, unspecified (Y/N) | ICD-10-CM | P229 |
| Extremely low birth weight newborn, 500-749 grams (Y/N) | ICD-10-CM | P0702 |
| Extremely low birth weight newborn, 750-999 grams (Y/N) | ICD-10-CM | P0703 |
| Other low birth weight newborn, 1000-1249 grams (Y/N) | ICD-10-CM | P0714 |
| Other low birth weight newborn, 1250-1499 grams (Y/N) | ICD-10-CM | P0705 |
| Other low birth weight newborn, 1500-1749 grams (Y/N) | ICD-10-CM | P0706 |
| Other low birth weight newborn, 1750-1999 grams (Y/N) | ICD-10-CM | P0707 |
| Other low birth weight newborn, 2000-2499 grams (Y/N) | ICD-10-CM | P0708 |
| Extreme immaturity of newborn, gestational age less than 23 completed weeks (Y/N) | ICD-10-CM | P0721 |
| Extreme immaturity of newborn, gestational age 23 completed weeks (Y/N) | ICD-10-CM | P0722 |
| Extreme immaturity of newborn, gestational age 24 completed weeks (Y/N) | ICD-10-CM | P0723 |
| Extreme immaturity of newborn, gestational age 25 completed weeks (Y/N) | ICD-10-CM | P0724 |
| Extreme immaturity of newborn, gestational age 26 completed weeks (Y/N) | ICD-10-CM | P0725 |
| Extreme immaturity of newborn, gestational age 27 completed weeks (Y/N) | ICD-10-CM | P0726 |
| Preterm newborn, gestational age 28 completed weeks (Y/N) | ICD-10-CM | P0731 |
| Preterm newborn, gestational age 29 completed weeks (Y/N) | ICD-10-CM | P0732 |
| Preterm newborn, gestational age 30 completed weeks (Y/N) | ICD-10-CM | P0733 |
| Preterm newborn, gestational age 31 completed weeks (Y/N) | ICD-10-CM | P0734 |
| Preterm newborn, gestational age 32 completed weeks (Y/N) | ICD-10-CM | P0735 |
| Preterm newborn, gestational age 33 completed weeks (Y/N) | ICD-10-CM | P0736 |
| Preterm newborn, gestational age 34 completed weeks (Y/N) | ICD-10-CM | P0737 |
| Preterm newborn, gestational age 35 completed weeks (Y/N) | ICD-10-CM | P0738 |
| Preterm newborn, gestational age 36 completed weeks (Y/N) | ICD-10-CM | P0739 |
| Respiratory Ventilation, 24-96 Consecutive Hours (Y/N) | ICD-10-PCS | 5A1945Z |
| Assistance with Respiratory Ventilation, 24-96 Consecutive Hours, Continuous Positive Airway Pressure (Y/N) | ICD-10-PCS | 5A09457 |
| Assistance with Respiratory Ventilation, 24-96 Consecutive Hours, High Nasal Flow/Velocity (Y/N) | ICD-10-PCS | 5A0945A |
| Assistance with Respiratory Ventilation, 24-96 Consecutive Hours, Intermittent Positive Airway Pressure (Y/N) | ICD-10-PCS | 5A09458 |
| Respiratory Ventilation, Greater than 96 Consecutive Hours (Y/N) | ICD-10-PCS | 5A1955Z |
| Assistance with Respiratory Ventilation, Greater than 96 Consecutive Hours, Continuous Positive Airway Pressure (Y/N) | ICD-10-PCS | 5A09557 |
| Assistance with Respiratory Ventilation, Greater than 96 Consecutive Hours, High Nasal Flow/Velocity (Y/N) | ICD-10-PCS | 5A0955A |
| Assistance with Respiratory Ventilation, Greater than 96 Consecutive Hours, Intermittent Positive Airway Pressure (Y/N) | ICD-10-PCS | 5A09558 |
| Respiratory Ventilation, Less than 24 Consecutive Hours (Y/N) | ICD-10-PCS | 5A1935Z |
| Assistance with Respiratory Ventilation, Less than 24 Consecutive Hours, Continuous Positive Airway Pressure (Y/N) | ICD-10-PCS | 5A09357 |
| Assistance with Respiratory Ventilation, Less than 24 Consecutive Hours, High Nasal Flow/Velocity (Y/N) | ICD-10-PCS | 5A0935A |
| Assistance with Respiratory Ventilation, Less than 24 Consecutive Hours, Intermittent Positive Airway Pressure (Y/N) | ICD-10-PCS | 5A09358 |
| Bypass Trachea to Cutaneous with Tracheostomy Device, Open Approach (Y/N) | ICD-10-PCS | 0B110F4 |
| Extracorporeal Oxygenation, Membrane, Peripheral Veno-arterial (Y/N) | ICD-10-PCS | 5A1522G |
| Insertion of Endotracheal Airway into Trachea, Via Natural or Artificial Opening (Y/N) | ICD-10-PCS | 0BH17EZ |
| Insertion of Endotracheal Airway into Trachea, Via Natural or Artificial Opening Endoscopic (Y/N) | ICD-10-PCS | 0BH18EZ |
| Insertion of Feeding Device into Stomach, Open Approach (Y/N) | ICD-10-PCS | 0DH60UZ |
| Introduction of Nitric Oxide Gas into Respiratory Tract, Via Natural or Artificial Opening (Y/N) | ICD-10-PCS | 3E0F7SD |
| Introduction of Other Gas into Respiratory Tract, Via Natural or Artificial Opening (Y/N) | ICD-10-PCS | 3E0F7SF |
| Introduction of Other Therapeutic Substance into Respiratory Tract, Via Natural or Artificial Opening (Y/N) | ICD-10-PCS | 3E0F7GC |
| Measurement of Arterial Saturation, Peripheral, External Approach (Y/N) | ICD-10-PCS | 4A03XR1 |
| Measurement of Arterial Saturation, Peripheral, Percutaneous Approach (Y/N) | ICD-10-PCS | 4A033R1 |
| Occlusion of Ductus Arteriosus with Extraluminal Device, Open Approach (Y/N) | ICD-10-PCS | 02LR0CT |
| Occlusion of Ductus Arteriosus with Intraluminal Device, Percutaneous Approach (Y/N) | ICD-10-PCS | 02LR3DT |
| Occlusion of Ductus Arteriosus, Open Approach (Y/N) | ICD-10-PCS | 02LR0ZT |
| Respiratory Ventilation, 24-96 Consecutive Hours (Y/N) | ICD-10-PCS | 5A1945Z |
| Respiratory Ventilation, Greater than 96 Consecutive Hours (Y/N) | ICD-10-PCS | 5A1955Z |
| Respiratory Ventilation, Less than 24 Consecutive Hours (Y/N) | ICD-10-PCS | 5A1935Z |
| Respiratory Ventilation, Single, Nonmechanical (Y/N) | ICD-10-PCS | 5A19054 |
| NB RESUSCITATION (Y/N) | CPT | 99465 |
