# Supplementary material for: An algorithm to identify less invasive surfactant administration using a real-world database of preterm infants
Source: PLoS One. 2026 Apr 15;21(4):e0345768. doi: 10.1371/journal.pone.0345768 (PMC13082626; doi:10.1371/journal.pone.0345768)
Supplement: S2 Table — (DOCX) [file pone.0345768.s003.docx]

**Supplemental Table 2. Demographic characteristics for testing cohort**

|  |  | Testing Cohort (N = 379) | |  |
| --- | --- | --- | --- | --- |
| **Characteristic** | Total, N=379 | Non-LISA, N = 238 | LISA, N = 141 | p-value*^1^* |
| **Sex, n (%)** |  |  |  | 0.6 |
| *Female* | 149 (39.3%) | 96 (40.3%) | 53 (37.6%) |  |
| *Male* | 230 (60.7%) | 142 (59.7%) | 88 (62.4%) |  |
| **Multiple pregnancy, n (%)** | 79 (20.8%) | 43 (18.1%) | 36 (25.5%) | 0.084 |
| **Birth facility level, n (%)** |  |  |  | 0.7 |
| *1* | 4 (1.1%) | 2 (0.8%) | 2 (1.4%) |  |
| *2* | 56 (14.8%) | 37 (15.5%) | 19 (13.5%) |  |
| *3* | 319 (84.1%) | 199 (83.6%) | 120 (85.1%) |  |
| **Type of delivery, n (%)** |  |  |  | 0.4 |
| *Cesarean* | 292 (77.0%) | 180 (75.6%) | 112 (79.4%) |  |
| *Vaginal* | 87 (23.0%) | 58 (24.4%) | 29 (20.6%) |  |
| **SGA, n (%)** | 33 (8.7%) | 21 (8.8%) | 12 (8.5%) | >0.9 |
| **LGA, n (%)** | 56 (14.8%) | 42 (17.6%) | 14 (9.9%) | 0.041 |
| **Gestational age (weeks), n (%)** |  |  |  | <0.001 |
| *23-27* | 131 (34.6%) | 103 (43.3%) | 28 (19.9%) |  |
| *28-31* | 118 (31.1%) | 62 (26.1%) | 56 (39.7%) |  |
| *32-36* | 130 (34.3%) | 73 (30.7%) | 57 (40.4%) |  |
| **Birth year, n (%)** |  |  |  | <0.001 |
| *2019* | 76 (20.1%) | 60 (25.2%) | 16 (11.3%) |  |
| *2020* | 70 (18.5%) | 55 (23.1%) | 15 (10.6%) |  |
| *2021* | 84 (22.2%) | 46 (19.3%) | 38 (27.0%) |  |
| *2022* | 65 (17.1%) | 34 (14.3%) | 31 (22.0%) |  |
| *2023* | 84 (22.2%) | 43 (18.1%) | 41 (29.1%) |  |
| **Final disposition, n (%)** |  |  |  | <0.001 |
| *Alive/Home* | 342 (90.2%) | 204 (85.7%) | 138 (97.9%) |  |
| *Death* | 37 (9.8%) | 34 (14.3%) | 3 (2.1%) |  |
| **ICD TTN, n (%)** | 42 (11.1%) | 19 (8.0%) | 23 (16.3%) | 0.013 |
| **ICD RDS, n (%)** | 352 (92.9%) | 222 (93.3%) | 130 (92.2%) | 0.7 |
| **ICD RDS other, n (%)** | 10 (2.6%) | 8 (3.4%) | 2 (1.4%) | 0.3 |
| **ICD RDS unspecified, n (%)** | 215 (56.7%) | 140 (58.8%) | 75 (53.2%) | 0.3 |
| **Apgar 5 score < 7, n (%)** | 97 (56.7%) | 85 (35.7%) | 12 (8.5%) | <0.001 |
| **Highest FiO2 pre-surfactant** |  |  |  |  |
| Mean (SD) | - | 64 (28) | 55 (24) | 0.002 |
| Median (IQR) | - | 60 (40, 100) | 50 (39, 70) | 0.010 |
| Unknown | 21 | 21 | 0 |  |
| **Respiratory support pre-surfactant, n (%)** |  |  |  | <0.001 |
| CVENT | 59 (15.6%) | 59 (24.8%) | 0 (0.0%) |  |
| HFNC | 2 (0.5%) | 1 (0.4%) | 1 (0.7%) |  |
| HFV | 4 (1.1%) | 4 (1.7%) | 0 (0.0%) |  |
| NCPAP | 258 (68.1%) | 124 (52.1%) | 134 (95.0%) |  |
| NIPPV | 9 (2.4%) | 4 (1.7%) | 5 (3.5%) |  |
| Unknown | 47 (12.4%) | 46 (19.3%) | 1 (0.7%) |  |
| **Maternal age** |  |  |  |  |
| Mean (SD) | - | 32.7 (6.0) | 33.3 (5.7) | 0.33 |
| Median (IQR) | - | 32.6 (28.7, 36.9) | 33.4 (30.2, 37.1) | 0.2 |
| Unknown | 1 | 1 | 0 |  |
| **Maternal race/ethnicity, n (%)** |  |  |  | 0.6 |
| *Asian* | 67 (17.7%) | 37 (15.5%) | 30 (21.3%) |  |
| *Black* | 56 (14.8%) | 35 (14.7%) | 21 (14.9%) |  |
| *Hispanic* | 108 (28.5%) | 72 (30.3%) | 36 (25.5%) |  |
| *Other/Missing* | 19 (5.0%) | 11 (4.6%) | 8 (5.7%) |  |
| *White* | 129 (34.0%) | 83 (34.9%) | 46 (32.6%) |  |
| **Antenatal steroid, n (%)** | 254 (67.0%) | 158 (66.4%) | 96 (68.1%) | 0.7 |
|  |  | | | |

*^1^*Pearson's Chi-squared test; Fisher's exact test; Wilcoxon rank sum test; Fisher's Exact Test for Count Data with simulated p-value (based on 2000 replicates)
