# Supplementary material for: An algorithm to identify less invasive surfactant administration using a real-world database of preterm infants
Source: PLoS One. 2026 Apr 15;21(4):e0345768. doi: 10.1371/journal.pone.0345768 (PMC13082626; doi:10.1371/journal.pone.0345768)
Supplement: S3 Table — (DOCX) [file pone.0345768.s004.docx]

Supplemental Table 3. Algorithm performance in training cohort overall and by gestational age using maximized specificity cut point

| Statistic | Overall | GA ≥34 weeks | GA ≥34 weeks |
| --- | --- | --- | --- |
| Number of infants (N) | 884 | 725 | 159 |
| Sensitivity, % (95% CI) | 41.1 (35.7–46.7) | 40.5 (34.3–46.9) | 43.2 (31.8–55.3) |
| Specificity, % (95% CI) | 99.1 (97.9–99.7) | 99.4 (98.2–99.9) | 97.7 (91.8–99.7) |
| Positive predictive value, % (95% CI) | 96.4 (91.6–98.5) | 97.1 (91.4–99.1) | 94.1 (79.9–98.5) |
| Negative predictive value, % (95% CI) | 74.7 (72.4–76.4) | 76.4 (74.5–78.2) | 66.4 (61.8–70.7) |
| Accuracy, % (95% CI) | 78.1 (75.2–80.7) | 79.3 (76.2–82.2) | 72.3 (64.7–79.1) |
| Positive likelihood ratio | 46.3 | 64.5 | 18.4 |
| Negative likelihood ratio | 0.59 | 0.60 | 0.58 |
| Estimated disease prevalence, % (95% CI) | 36.3 (33.1–39.6) | 34.1 (30.6–37.7) | 46.5 (38.6–54.6) |
