# Supplementary material for: An algorithm to identify less invasive surfactant administration using a real-world database of preterm infants
Source: PLoS One. 2026 Apr 15;21(4):e0345768. doi: 10.1371/journal.pone.0345768 (PMC13082626; doi:10.1371/journal.pone.0345768)
Supplement: S4 Table — (DOCX) [file pone.0345768.s005.docx]

Supplemental Table 4. Algorithm performance in training cohort overall and by gestational age using Youden’s cut point

| Statistic | Overall | GA ≥34 weeks | GA ≥34 weeks |
| --- | --- | --- | --- |
| Number of infants (N) | 884 | 725 | 159 |
| Sensitivity, % (95% CI) | 71.3 (66.1–76.2) | 73.3 (67.3–78.7) | 64.9 (52.9–75.6) |
| Specificity, % (95% CI) | 90.6 (87.8–92.9) | 91.0 (88.1–93.4) | 88.2 (79.4–94.2) |
| Positive predictive value, % (95% CI) | 84.2 (77.6–84.9) | 80.8 (75.8–85.0) | 82.8 (72.7–89.8) |
| Negative predictive value, % (95% CI) | 84.8 (82.4–86.9) | 86.4 (83.2–89.0) | 74.3 (67.0–79.9) |
| Accuracy, % (95% CI) | 83.6 (80.9–86.0) | 84.9 (82.2–87.5) | 77.4 (70.1–83.6) |
| Positive likelihood ratio | 7.6 | 8.2 | 5.5 |
| Negative likelihood ratio | 0.32 | 0.29 | 0.40 |
| Estimated disease prevalence, % (95% CI) | 36.3 (33.1–39.6) | 34.1 (30.6–37.7) | 46.5 (38.6–54.6) |
