# Supplementary material for: An algorithm to identify less invasive surfactant administration using a real-world database of preterm infants
Source: PLoS One. 2026 Apr 15;21(4):e0345768. doi: 10.1371/journal.pone.0345768 (PMC13082626; doi:10.1371/journal.pone.0345768)
Supplement: S5 Table — (DOCX) [file pone.0345768.s006.docx]

Supplemental Table 5. Algorithm validation in testing + 2024 birth cohort overall and by gestational age using Youden’s cut point

| Statistic | Overall | GA ≥34 weeks | GA ≥34 weeks |
| --- | --- | --- | --- |
| Number of infants (N) | 622 | 494 | 128 |
| Sensitivity, % (95% CI) | 74.0 (68.0–79.4) | 70.7 (63.7–77.0) | 85.5 (73.3–93.5) |
| Specificity, % (95% CI) | 79.8 (75.4–83.7) | 82.5 (77.8–86.6) | 68.5 (56.6–78.9) |
| Positive predictive value, % (95% CI) | 70.5 (65.9–74.8) | 71.8 (66.2–76.8) | 67.1 (58.9–74.5) |
| Negative predictive value, % (95% CI) | 82.3 (79.1–85.3) | 81.9 (78.1–84.8) | 86.2 (76.4–92.4) |
| Accuracy, % (95% CI) | 77.5 (74.0–80.7) | 77.9 (74.0–81.5) | 75.8 (67.4–82.9) |
| Positive likelihood ratio | 3.66 | 4.04 | 2.71 |
| Negative likelihood ratio | 0.33 | 0.36 | 0.21 |
| Estimated disease prevalence, % (95% CI) | 39.6 (35.7–43.5) | 38.7 (34.4–43.1) | 42.9 (34.3–52.0) |
